# Heterogeneity in the development of proactive and reactive aggression in childhood: Common and specific genetic - environmental factors

**DOI:** 10.1371/journal.pone.0188730

**Published:** 2017-12-06

**Authors:** Stéphane Paquin, Eric Lacourse, Mara Brendgen, Frank Vitaro, Ginette Dionne, Richard Ernest Tremblay, Michel Boivin

**Affiliations:** 1 Research Unit on Children’s Psychosocial Adjustment, Ste-Justine Hospital Research Center, Montreal, Canada; 2 Department of Sociology, Université de Montréal, Montreal, Canada; 3 Department of Psychology, Université du Québec à Montréal, Montreal, Canada; 4 School of Psychoeducation, Université de Montréal, Montreal, Canada; 5 Department of Psychology, Université Laval, Montreal, Canada; 6 Departments of Pediatrics and Psychology, Université de Montréal, Montreal, Canada; 7 School of Public Health, Physiotherapy and Populations Sciences, University College Dublin, Dublin, Ireland; 8 Institute of Genetic, Neurobiological and Social Foundations of Child Development, Tomsk State University, Tomsk, Russian Federation; National Eye Institute, UNITED STATES

## Abstract

**Background:**

Few studies are grounded in a developmental framework to study proactive and reactive aggression. Furthermore, although distinctive correlates, predictors and outcomes have been highlighted, proactive and reactive aggression are substantially correlated. To our knowledge, no empirical study has examined the communality of genetic and environmental underpinning of the development of both subtypes of aggression. The current study investigated the communality and specificity of genetic-environmental factors related to heterogeneity in proactive and reactive aggression’s development throughout childhood.

**Methods:**

Participants were 223 monozygotic and 332 dizygotic pairs. Teacher reports of aggression were obtained at 6, 7, 9, 10 and 12 years of age. Joint development of both phenotypes were analyzed through a multivariate latent growth curve model. Set point, differentiation, and genetic maturation/environmental modulation hypotheses were tested using a biometric decomposition of intercepts and slopes.

**Results:**

Common genetic factors accounted for 64% of the total variation of proactive and reactive aggression’s intercepts. Two other sets of uncorrelated genetic factors accounted for reactive aggression’s intercept (17%) on the one hand, and for proactive (43%) and reactive (13%) aggression’s slopes on the other. Common shared environmental factors were associated with proactive aggression’s intercept (21%) and slope (26%) and uncorrelated shared environmental factors were also associated with reactive aggression’s slope (14%). Common nonshared environmental factors explained most of the remaining variability of proactive and reactive aggression slopes.

**Conclusions:**

A genetic differentiation hypothesis common to both phenotypes was supported by common genetic factors associated with the developmental heterogeneity of proactive and reactive aggression in childhood. A genetic maturation hypothesis common to both phenotypes, albeit stronger for proactive aggression, was supported by common genetic factors associated with proactive and reactive aggression slopes. A shared environment set point hypothesis for proactive aggression was supported by shared environmental factors associated with proactive aggression baseline and slope. Although there are many common features to proactive and reactive aggression, the current research underscores the advantages of differentiating them when studying aggression.

## Background

Aggression is a broadly defined construct that covers many normative and pathological behaviors that can differ throughout development. Heterogeneity in forms (e.g., physical, relational) and functions (i.e., proactive, reactive) are noted in many previous studies [1, 2]. The consideration of both forms and functions of aggression have inspired researchers to propose a dimensional conceptualization of aggression [3, 4]. With respect to functions, the distinction between proactive aggression (aggression that is directed toward others with an intent to harm, PA) and reactive aggression (aggression that is a defensive behavior against provocations or threats, RA) was inspired by studies of offensive and defensive aggression in animals [3]. The proactive and reactive concepts were later proposed by Dodge & Coie [2] who developed two scales based on factor analysis of twelve items chosen to capture Bandura [5] and Berkowitz [6] respective notion of aggression. Bandura [5] stated that aggression could be understood as a learned instrumental behavior intended to obtain a reward or reach an objective. He showed that aggression could be initiated by imitation [7] and learned through operant conditioning. According to this theory, the positive expected outcome could be the main driving force of aggressive behavior. In contrast, Berkowitz’s frustration-aggression model stated that aggression was an angry reaction to frustration [8, 9]. In the frustration-aggression model, threat, goal blocking and anger are all potential triggers to aggressive behavior. These theories refer to multiple functions or motivations behind individual’s aggressive behaviors [9–11]. Essentially, Bandura’s social learning approach is cited to explain proactive, instrumental aggression whereas the frustration-aggression model is used to describe reactive aggressive behaviors that are generally more impulsive or anger-driven and triggered by perception of contextual cues, such as an apparent threat.

Studies of the development of aggression suggest that physical aggression against others is the first form to be expressed in early life. Developmental studies have shown that physical aggression peaks between 2 and 4 years of age [12] and gradually decreases until adulthood [13, 14]. Researchers have suggested that the development of cognitions and language skills with more frequent interactions with peers during early childhood led to the onset and increase of other more subtle forms of aggression, such as relational aggression [15]. It has also been hypothesized that aggression starts to serve different functions during childhood as cognitive skills develop. Researchers have shown evidence that instrumental aggression is observable by 6 months of age [16, 17]. Childhood is thus a suitable developmental period to study the development of the functions of aggression because sufficient development is needed to enable planning of an aggressive act such as PA or voluntary control over RA. Surprisingly, individual development of PA and RA has rarely been investigated. The few existing studies are consistent with research on general aggression and show a small declining tendency for both functions of aggression through childhood [18, 19] and into adolescence [20].

### Meaningful differences

The conceptual distinction of PA and RA have often been addressed because the two behaviors are highly correlated when measured concurrently (between .68 - .71) [21–23]. Nevertheless, the distinction is supported by research showing that PA and RA are associated with specific cognitive, temperamental and socialization predictors and outcomes. For example, during the cognitive process of response in social interactions, PA children selected instrumental goals and were more confident in the use of aggression compared to reactive children [24]. Callous-unemotional traits have also been associated with PA [25] along other psychopathic traits [26]. Socialization factors are also differently related to PA and RA. Some specifically associated with PA are parents’ endorsement of aggressive behavior as an adequate goal-directed behavior [27], lack of parental discipline and monitoring [28–32], affiliation with deviant peers [33, 34] and popularity status [35]. In comparison, RA has been associated with hostile attributions toward potential sources of threats or pain [36, 37], low effortful control [38, 39], and generally to deficits in executive functioning [40, 41]. RA has also been associated with traits such as negative emotionality [42], anxiety [26] and anger [43]. Researchers also suggested that the endocrine system would likely be involved in the regulation of RA through its effect on the modulation of impulsivity [44]. Socialization factors that might increase or decrease RA include lack of parental warmth and care [36], physical or emotional abuse and neglecting parents [30, 33, 45], and peer victimization [46, 47].

Behavioral genetic studies have shown that aggressive behavior is moderately to strongly related to genetic factors (A). Environmental factors that are shared by twins (C) and environmental factors unique to each twin (E) seem to play a lesser role [48–51]. However, few genetically informed studies have specifically examined the genetic and environmental architecture of PA and RA. In children aged 9–10 years, Baker, Raine [52] found that genetic factors accounted for 45% of the variance of teacher-rated PA, but only 20% of the variance of RA. They also found significant shared environmental factors, explaining respectively 14% and 43%, of the variance of teacher-rated PA and RA. Another cross-sectional study of 6 years-old children from Brendgen et al. found that genetic factors accounted for 41% of PA and 39% of RA’s variance [53] while the remaining phenotypic variance was associated with nonshared environmental factors that also include measurement errors.

### Explanation for the overlap

Some factors seem less clearly associated with a specific function. Actually, some of the factors identified in the literature as specific to one function are based on residualized correlations (associated with the residual of a function or conditional on the other function). Scholars have suggested interpreting these residualized associations with caution [54] because residuals correspond to different things based on the statistical model that is executed. Besides, the validity of these residualized constructs is not demonstrated once the common variance with the other form of aggression is partialed out. For example, while psychopathy is generally only associated with PA when using residualized scores, the use of raw scores show that most components of psychopathy are associated with both PA and RA [26, 55]. Also, researchers have recently proposed that neurotransmitters from the aminergic system could be involved in the regulation of both PA and RA [44] through their role in response to stress and rewards. Finally, an influential study from Little, Henrich (4) suggests the overlap between PA and RA is likely due to the form of aggression captured by these measures, namely the overt form of aggression. Yet, none of the above studies considered the possibility of common genetic influences. Furthermore, a previously cited study found the correlation between PA and RA’s genetic factors was about .87 and this correlation was entirely accounted for by their joint overlap with physical aggression, a measure of the form of aggression [53]. This study suggests that PA and RA in children 6 years of age have substantial common genetic factors, yet this cross-sectional study can’t inform on genetic factors associated with interindividual variance of intraindividual development of PA and RA.

Longitudinal studies of aggression shows that the stability of aggression through childhood has a relatively high level of heritability; around 60% to 80% [51]. Few behavioral genetic studies examined specifically PA and RA at two or more time points and provided information regarding the relative influence of genetic and environmental factors during childhood and the beginning of adolescence. One study used parent-rated aggression scores at two time points (ages 9–10 and 11–14 years) allowing the estimation of common genetic and environmental factors associated with the stability of PA and RA [56]. The study show that common genetic factors explained 63% of the stability of PA and 80% of the stability of RA. Common nonshared environmental factors explained the remaining 37% and 20% of the stability of PA and RA. In line with the Brendgen’s study, their results suggest genetic factors are substantially associated with interindividual stability between 9–10 and 11–14 years of age, however, it does not inform on the variations in the baseline level of aggression and on its change over time.

Finally, in another study, Paquin, Lacourse (57) investigated PA and RA through childhood (from age 6 to age 12 years) using a time-specific general latent factor model. Their model allowed to simultaneously investigate the genetic and environmental factors associated with latent aggression factors (measured by PA and RA) as well as the genetic and environmental underpinnings of their residual variances at each time point. They showed that the latent aggression factors at each age were influenced by common genetic factors but with a decreasing magnitude through time. Innovative genetic factors at 7, 9 and 10 years were associated with latent aggression factors for shorter time spans. Thus, Paquin et al. study showed evidence that some genetic factors are common to PA and RA and have persistent associations with aggression states through childhood. Together, the results from the Tuvblad et al. and the Paquin et al. studies suggest common genetic factors have a persistent effect on PA and RA states through childhood [57] and at the beginning of adolescence [56]. These studies also suggest that nonshared environmental factors have influences of shorter duration.

To conclude, few studies have described and explained the intraindividual developmental process of PA and RA, and none have examined their joint development. Furthermore, while the literature has established significant overlap between PA and RA, few have examined the possibility of common genetic factors, and none have looked at the genetic and environmental factors associated with interindividual differences in development [58].

### Objectives

**Fig 1:**
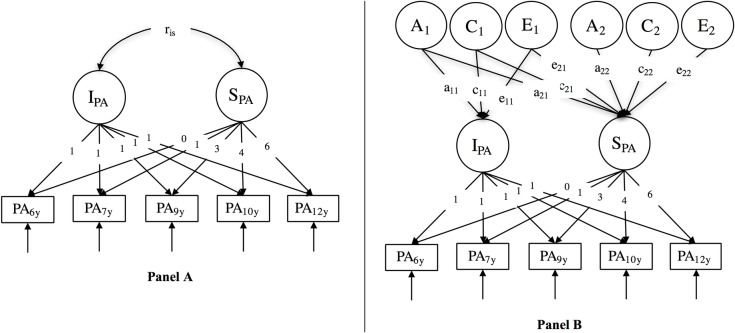
**Latent growth curve model (panel A) and biometric latent growth curve model (panel B) of proactive aggression.** Proactive aggression is illustrated here, the same models are used for reactive aggression. Naming scheme of the parameters: The letter refers to the biometric component, the first number refers to the destination of an arrow, and the last number to the origin of an arrow. For example, a_21_ indicate a link from the 1^st^ genetic component to the 2^nd^ latent variable (here a slope).

To address these issues, the objective of the present study was to use latent growth curve models to examine the genetic and environmental architecture of the development of PA and RA through childhood (i.e., from age 6 through age 12 years). Latent growth curve models summarize the developmental course of PA and RA with two main parameters: the intercept (baseline level) and the slope (systematic developmental change) (Fig 1A). Furthermore, with a genetically informed design, a *biometric* latent growth curve model can partition the interindividual variance and covariance of the intercepts and slopes parameters into genetic (A), shared (C) and nonshared (E) environmental factors (Fig 1B) [48, 59, 60].

The biometric decomposition of the relation between baseline level and developmental change allows to test three hypotheses for each biometric component (see Table 1 for a detailed presentation of these hypotheses). First, the set point hypothesis implies that the same A, C or E factors are associated with the baseline level and developmental change of aggressive behavior. This hypothesis implies that genetic or environmental factors present at 6 years of age allow to describe the whole development of PA or RA through childhood. Second, the differentiation hypothesis denotes that the variation in the baseline and developmental change is weakly correlated. Thus, A, C or E factors associated with variation in the baseline level of aggression are not related to the developmental change. The third hypothesis, the genetic maturation or environment modulation, indicates that A, C or E factors associated with the interindividual variation of developmental change are independent of factors associated with the variation in baseline levels. In this last hypothesis, factors that can describe change in aggression through childhood are weakly related to the ones describing variation in baseline levels. We can note that the set point and the differentiation hypotheses are mutually exclusive while the maturation/modulation hypothesis can be supported simultaneously with either the set point or the differentiation hypotheses.

**Table 1:** Hypotheses suggested by the biometric decomposition of intercepts (baseline level) and slopes (developmental change) of PA and RA.

Biometric components	Hypotheses	Parameters of interest^1^	Theoretical interpretation
A	Set point	a_11_ ≠ 0 & a_21_ ≠ 0	The same genetic factors are associated with variation in baseline level and variation in developmental change
	Differentiation	a_11_ ≠ 0 & a_21_ = 0	Variation in baseline level is associated with genetic factors that are independent of developmental change
	Maturation	a_22_ ≠ 0	Variation in developmental change is associated with genetic factors that are independent from the baseline level
C, E	Set point	c_11_ ≠ 0 & c_21_ ≠ 0, e_11_ ≠ 0 & e_21_ ≠ 0	The same shared or nonshared environmental factors are associated with variation in baseline level and variation in developmental change
	Differentiation	c_11_ ≠ 0 & c_21_ = 0, e_11_ ≠ 0 & e_21_ = 0	Variation in baseline level is associated with shared or nonshared environmental factors that are independent of developmental change
	Modulation	c_22_ ≠ 0, e_22_ ≠ 0	Variation in developmental change is associated with shared or nonshared environmental factors that are independent from the baseline level

^1^ Parameters are illustrated in Fig 1B.

We first performed separate biometric latent growth curve model of the development of PA and RA from ages 6 to 12 years. Our main objective was to examine if the genetic and environmental hypotheses identified in univariate models of development were common to PA and RA or specific to each subtype of aggression. This was addressed with the multivariate extension of the latent growth curve model. By applying a biometric decomposition of the covariances in baseline levels and systematic developmental changes of PA and RA, we could examine whether set point, differentiation or maturation/modulation hypotheses were common or specific to PA and RA.

## Methods

### Sample

Participants were part of the ongoing longitudinal Québec Study of Newborn Twins, which comprised 667 twin pairs (254 monozygotic [MZ] and 413 dizygotic [DZ] pairs) who were first evaluated at the age of 5 months [61]. The sample comprises 568 boys and 542 girls distributed in 115 pairs of MZ boys, 108 pairs of MZ girls, 88 pairs of same-sex DZ boys, 82 pairs of same-sex DZ girls, and 162 pairs of opposite-sex DZ. The zygosity was assessed at the age of 18 months on the basis of physical resemblance via the Zygosity Questionnaire for Young Twins [62]. For a subsample of these twin pairs (*n* = 123), a DNA sample was analyzed using 8–10 highly polymorphous genetic markers [63]. The comparison of zygosity based on the similarity of these genetic markers with zygosity based on physical resemblance revealed a 94% correspondence rate, which is similar to rates obtained in older twin samples. Eighty-four percent of the families were of European descent, 3% were of African descent, 2% were of Asian descent, and 2% were Native North Americans. The remaining families (9%) did not provide ethnicity information. The demographic characteristics of the twin families were compared with those of a sample of single births representative of the large urban centers in the province of Québec when the children were 5 months of age [64]. The results showed that the same percentage (95%) of parents in both samples lived together at the time of the birth of their child(ren); 44% of the twins compared with 45% of the singletons were the firstborn children in the family; 66% of the mothers and 60% of the fathers of the twins and 66% of the mothers and 63% of the fathers of the singletons were between 25 and 34 years of age; 17% of the mothers and 14% of the fathers of the twins had not finished high school compared with 12% (mothers) and 14% (fathers) of the singletons; the same proportion of mothers (28%) and fathers (27%) in both samples held a university degree; 83% of the twin families and 79% of singleton families were employed; 10% of the twin families and 9% of the singleton families received social welfare or unemployment insurance; and finally, 30% of the twin families and 29% of the singleton families had an annual total income of less than $30,000, 44% (42%) had an annual total income between $30,000 and $59,999, and 27% (29%) had an annual total income of more than $60,000. These results indicate extremely similar socio-demographic profiles in the twin sample and the representative sample of single births.

The longitudinal sample was first assessed at 5 months and then annually to 12 years. The present study is based on teacher reports of PA and RA when participants were 6, 7, 9, 10 and, 12 years old, respectively. We used 555 twin pairs (223 MZ and 332 DZ pairs) for whom at least one of the twins had at least one valid data point on either the PA or RA items. Compared to the pairs used in our analyses, the pairs lost to attrition had lower familial revenues, fathers had a slightly higher education level and were more often DZ pairs. The pairs lost to attrition did not differ on sex, opposition or hyperactivity levels measured at 6 years of age. The maximum likelihood estimator used in the analyses makes use of all available data and treats the data as missing at random.

All instruments were administered in either English or French, depending on the language spoken by the kindergarten teachers (see description of measures below). Following the procedure suggested by Vallerand [65], instruments that were administered in French but were originally written in English were first translated into French and then translated back into English. Bilingual judges verified the semantic similarity between the back-translated items and the original items in the questionnaire. Data collections took place in the spring of the school year to ensure that teachers were well acquainted with the children in their class. Prior to data collection, written consent from the parents of all the children was obtained. The instruments were approved by each school board and by the Ste-Justine Hospital Research Ethics Board.

### Measures

PA and RA were assessed with the teacher-rated scale developed by Dodge and Coie (2). Teacher ratings of primary school children’s aggression have been found to correlate well with peer ratings of aggression (i.e., where all classmates evaluate each other’s behavior), with correlations between .78 and .83 [66]. Also, the fact that teachers changed from one year to the next prevented children’s aggression to be systematically evaluated by the same teacher, thus further reducing the risk of consistent rater bias on children’s aggression ratings. The instrument is comprised of three proactive (gets others to gang up on a peer; threatens and bullies others; uses physical force to dominate) and three reactive (overreacts angrily to accidents; blames others in fight; when teased, strikes back) aggression items. A fourth reactive aggression item (reacts in an aggressive manner when contradicted) was added to assess the extent to which children behave in a reactively aggressive manner even in a rather benign, less provocative context. Responses were given on a three-point scale (0 = *never*, 1 = *sometimes*, 2 = *often*). Both scales were computed by averaging their respective items. The average was computed for cases with at least 2 out of 3 valid values for PA and 3 out of 4 valid values for RA. We then multiplied the means by the number of items in each scale. This allows the scale to have the same minimum and maximum for each case without consideration of the number of valid items per case. The internal consistency of the total scale for each year was acceptable in the present sample with Cronbach’s *α* ranging from .68 to .78 for teacher-rated proactive aggression and .87 to .89 for teacher-rated reactive aggression. All scales were log-transformed before inclusion in the data analysis.

### Analyses

At first, we performed two univariate latent growth curve models for PA and RA separately. We use the expression *univariate* latent growth curve models to distinguish these analyses from the following *multivariate* latent growth curve model that simultaneously includes both PA and RA. For the univariate analyses, we first fitted a latent phenotypic growth curve model for PA and RA to obtain estimates of intercepts and slopes. In these phenotypic models, the covariances between latent growth parameters as well as covariances between time-specific residuals could vary across zygosity. Second, we executed a biometric latent growth curve model where the covariances between latent growth parameters were replaced by a Cholesky decomposition of the variance/covariance matrix of these parameters (Fig 1B). We adopted a conservative approach to the modeling of the biometric components. To avoid over- or underestimating the importance of each biometric latent factor, we restricted latent genetic (A), shared (C) and nonshared (E) environmental factors to 0 only when their factor loadings were estimated to be close to zero and not based on likelihood ratio tests as is common practice in the behavior genetics literature.

**Fig 2:**
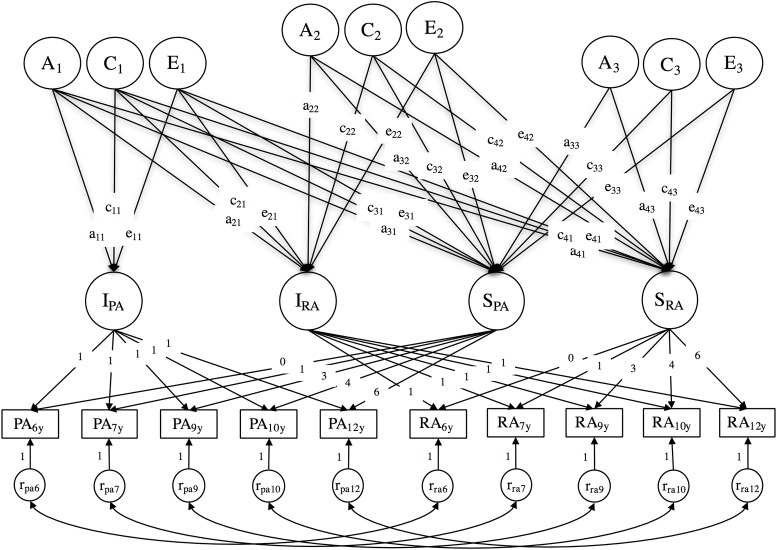
Cholesky decomposition of growth parameters in a bivariate latent growth curve model of proactive and reactive aggression. Naming scheme of the parameters: The letter refers to the biometric component, the first number refers to the destination of an arrow, and the last number to the origin of an arrow. For example, a_31_ indicate a link from the 1^st^ genetic component to the 3^rd^ latent variable (here, PA’s slope).

For our second objective, we used a multivariate latent growth curve model to test if PA and RA are related at the level of their respective growth parameters. It is a model that can be extended to test how common factors are associated with covariance of the baseline level and developmental change [67]. We thus extended the multivariate latent growth curve model to a biometric version. A Cholesky decomposition of the variance/covariance matrix of baseline levels and developmental changes of PA and RA was used to model their genetic and environmental architecture (Fig 2). The following sequence was used for the Cholesky decomposition: baseline of PA, baseline of RA, change in PA, and change in RA. We used that sequence because in a previous study with the same data, a common latent factor model showed that PA had very little variance left after accounting for the common variance with RA [57]. In that study, the latent aggression factors accounted for most of the genetic variability of PA while RA’s residuals had between 12% and 22% of specific genetic factors.

The first genetic factor (A_1_) in this Cholesky decomposition explains variance in the first growth parameter (here, the intercept of PA) as well as the genetic variance common between that first growth parameter and all following parameters. The second latent genetic factor (A_2_) explains genetic variance of the second growth parameter (here, the intercept of RA) that is independent of the precedent growth parameter (here, the intercept of PA) as well as genetic variance common between that second growth parameter and all next growth parameters in the sequence. The same interpretation holds for genetic factors located further in the sequence, as well as for the shared and nonshared environmental factors. Analyses were performed using maximum likelihood in Mplus v7.1 (Muthén, 1998–2012) with bootstrapped standard errors [68] and log-transformed dependent variables. Mplus syntax used can be found at the Open Science Framework (https://osf.io/hcqb3/). Reproduction can be executed with the full sample covariance matrix with means and standard errors found in S1 Table of the supporting information.

## Results

Means, bivariate phenotypic correlations and between-phenotype correlations are presented in Table 2. The means of PA followed a slow decline from 6 to 12 years of age (from *M*_MZ_ = .36; *M*_DZ_ = .39 to *M*_MZ_ = *M*_DZ_ = .23), similarly, RA scores also decreased (from *M*_MZ_ = .45; *M*_DZ_ = .54 to *M*_MZ_ = .34; *M*_DZ_ = .33). The phenotypic correlations were moderate at one year intervals and became stronger with age (from *r* = .38 to 46 for PA, and from *r* = .45 to .56 for RA), despite the fact that the participants had different teachers from one year to the next. As the intervals between the years increased, the correlations decreased, but remained significant even after a six-year interval (*r* = .20 for PA, and *r* = .31 for RA). The within-time phenotypic correlations between PA and RA at each assessment were high, ranging from *r* = .56 to *r* = .62.

**Table 2:** Means, phenotypic correlations, between-subtype correlations and intraclass correlations.

		6 years	7 years	9 years	10 years	12 years
Means (SD)						
Proactive	MZ	.36 (.49)	.34 (.50)	.33 (.54)	.28 (.48)	.23 (.43)
	DZ	.39 (.53)	.34 (.50)	.32 (.51)	.33 (.53)	.23 (.43)
Reactive	MZ	.45 (.59)	.48 (.61)	.47 (.63)	.50 (.61)	.34 (.54)
	DZ	.54 (.64)	.48 (.60)	.48 (.62)	.46 (.60)	.33 (.52)
Phenotypic correlations						
Proactive	7 years	.38				
	9 years	.22	.44			
	10 years	.22	.29	.46		
	12 years	.20	.35	.36	.46	
Reactive	7 years	.45				
	9 years	.37	.50			
	10 years	.32	.45	.56		
	12 years	.31	.43	.50	.52	
Phenotypic correlations between subtypes						
		Proactive				
Reactive	6 years	.59	.37	.23	.26	.22
	7 years	.28	.62	.35	.29	.29
	9 years	.29	.42	.62	.45	.40
	10 years	.21	.33	.44	.61	.43
	12 years	.16	.37	.34	.41	.56
MZ / DZ intraclass correlations						
Proactive	6 years		.50 / .28			
	7 years	.33 / .21	.37 / .23			
	9 years	.18 / .06	.38 / .17	.56 / .20		
	10 years	.21 / .10	.29 / .15	.39 / .17	.42 / .17	
	12 years	.12 / .14	.27 / .06	.38 / .18	.46 / .14	.43 / .24
Reactive	6 years	.52 / .28				
	7 years	.37 / .19	.51 / .25			
	9 years	.36 / .14	.44 / .22	.64 / .21		
	10 years	.25 / .14	.40 / .19	.49 / .21	.56 / .22	
	12 years	.28 / .16	.23 / .18	.39 / .21	.40 / .24	.49 / .27

The intraclass correlations (correlation between twins of the same pair) are also presented in Table 2. These correlations were computed from the variance and covariance estimates obtained in a fully saturated model. Specifically, we divided the within pair covariance by the square root of the product of each twin variance [correlation = covariance_(twin1-twin2)_/sq(variance_twin1_*variance_twin2_)]. For both phenotypes, the MZ intraclass correlations were generally about twice as high as the DZ correlations, which suggests significant additive genetic effects. Some shared environment contribution is also suggested for PA at ages 6 and 7 years and for the stability of PA between those ages, as the MZ and DZ correlations were closer, relative to most intraclass correlations. It is important to note that the intraclass correlations are based on interindividual variance. Any genetic or environmental effects associated with baseline level or developmental change captured by the intercept and slope parameters of latent growth curve models cannot be predicted from these intraclass correlations.

### Multivariate latent growth curves

As stated in the Analyses section, we first performed two univariate latent growth curve models for each subtype of aggression: the latent phenotypic growth curve model and the biometric latent growth curve. The first was estimated to describe the phenotypic development of each aggression subtype and the second to estimate genetic and environmental factors associated with baseline levels and developmental changes of PA and RA. We also tested linear and quadratic forms for each phenotype and found that linear trend captured adequately the development of PA and RA. Because the results replicated very well in the multivariate latent growth curve model, we focus here on those and invite readers to consult the univariate results in the supplemental material. We show the fit statistics in S2A Table, standardized variance components in S2B Table, and unstandardized parameters estimates in S3 Table of the supplemental material.

For the multivariate part of the analyses, we also executed a phenotypic model followed by a biometric model. The respective fit indices of each model are shown in Table 3. Models had acceptable fit with CFI of .92 or above and RMSEA of .05. The phenotypic multivariate latent growth curve model showed developmental trends for PA and RA similar to their respective univariate latent growth curve models. PA had an intercept that was significantly different from zero (I_MZ_ = .37, CI 95% .32-.43; I_DZ_ = .39, CI 95% .35-.44) and a slight yet significant decline from 6 to 12 years of age for MZ and DZ pairs (S_MZ_ = -.02, CI 95% -.04—.01; S_DZ_ = -.02, CI 95% -.03—.01). A small covariance between the intercept and slope of PA was found for MZ and DZ pairs (cov_IS-MZ_ = -.02, CI 95% -.02—.001; cov_IS-DZ_ = -.01, CI 95% -.02—.002). RA also had an intercept that was significantly different from zero (I_MZ_ = .51, CI 95% .44-.58; I_DZ_ = .56, CI 95% .51-.62) and a slight significant decline from 6 to 12 years of age (S_MZ_ = -.02, CI 95% -.04—.005; S_DZ_ = -.03, CI 95% -.05—.02). The covariance between the intercept and slope of RA was also small and nonsignificant (cov_IS-MZ_ = -.01, CI 95% -.02-.001; cov_IS-DZ_ = -.01, CI 95% -.02-.001).

**Table 3:** Fit statistics for the multivariate latent growth model.

Model	*LL*	AIC	BIC	CFI	RMSEA
Phenotypic growth	-4607.58	9431.17	9897.62	.92	.05
Biometric growth	-4599.15	9416.30	9887.07	.93	.05

The biometric bivariate growth model provided information regarding our main objective, namely to what extent genetic and environmental factors were common to the development of PA and RA or specific to each phenotype. The results from the biometric multivariate latent growth model are shown in Table 4 (unstandardized parameter estimates can be found in S4 Table of the accompanying supporting material). A first common additive genetic factor (A_1_) captured, respectively, 64.4% and 63.5% of the intercept variances of PA and RA. Also, over the common genetic factors shared with PA’s intercept, a new additive genetic factor (A_2_) explained additional variance in the intercept of RA (around 16.8%). Shared environmental factors (C_1_) were also associated with PA’s intercept (21%), but those were not associated with RA. Nonshared environmental factors (E_1_) were also associated with both PA and RA’s intercept, but explained a smaller part of their variances (respectively, 15.6% and 9.7%). This suggests that the covariance between intercepts of PA and RA is mainly due to common genetic factors. Slopes of PA and RA were associated with genetic, shared and nonshared environmental factors. Genetic factors (A_3_) that were independent from the intercepts explained about 42.7% of the variation in PA’s slope and 12.9% of the variation in RA’s. Shared environmental factors associated with PA’s slope (26.2%) were mostly the same ones that also affected PA’s intercept (C_1_). On the other hand, RA’s slope was associated with two different sets of shared environmental factor (C_2_ and C_3_) that together explained about 26% of RA’s slope. The remaining variance of the slopes of PA and RA was mostly explained by nonshared environmental factors, some that were specific to RA’S slope (E_2_: 13.4%) and some that were shared by PA and RA’s slopes (E_3_). Those explained around 15.4% of PA’s slope variance and 33.1% or RA’s. It is worth noting that the residuals are time-specific in the model, thus, the nonshared environmental effects associated with the intercepts and slopes are free of measurement error.

**Table 4:** Standardized portion of the phenotypes variance associated with genetic, shared and nonshared environmental factors in the multivariate biometric latent growth curve model (%).

Parameter	A_1_	A_2_	A_3_	C_1_	C_2_	C_3_	E_1_	E_2_	E_3_
Intercept PA, I_PA_	**64.4**			**21.0**			**14.6**		
Intercept RA, I_RA_	**63.5**	*16*.*8*		**2.4**	**1.4**		**9.7**	6.2	
Slope PA, S_PA_	**3.9**	0	**42.7**	**26.2**	**1.5**	6.2	**4.1**	.1	**15.4**
Slope RA, S_RA_	**5.6**	0	**12.9**	**8.3**	**14.3**	*11*.*7*	**.7**	13.4	**33.1**

Note. Each row sum to 100%. Bold values are significant at the .05 level, and italic at the .10 level. Significance is based on confidence intervals from 10 000 boostrapped samples.

## Discussion

Aggression is a complex construct that encompasses many forms and functions which have multiple causes. PA and RA indeed share common factors which may be associated with the overt form of aggression [69] such as psychopathic traits or neurological functioning. In addition, PA and RA can also be associated with specific factors over the ones they have in common.

Our results suggest that baseline levels of PA and RA are partly influenced by common genetic factors, supporting a common genetic differentiation hypothesis. This finding could help understand the high correlation between PA and RA. It also brings support to the hypothesis that their high correlation could be due to the physical [53] or overt form of aggression [4]. This speculative argument is based on the finding that physical aggression has already been associated with strong genetic effects [48], especially in childhood [70]. The more complicated question is what are precisely those genetic influences. Our study cannot directly address this question but hypotheses that temperamental characteristics such as anxiety, anger or psychopathic traits [26] could partly explain the common variation at the baseline level of PA and RA is supported by our results. Also, researchers have recently proposed that neurotransmitters from the aminergic system could be involved in the regulation of both PA and RA [44] through their role in response to stress and rewards.

We found evidence of common genetic factors associated with developmental changes of PA and RA. Those genetic factors were different from the ones influencing their baseline levels, suggesting a common genetic maturation hypothesis. These genetic factors could be related to cognitive factors that are maturing during childhood and important for executive functioning and, indirectly, aggression [71]. Following Pingault, Rijsdijk [60], we suggest that components of executive functioning that mature through childhood, such as planning, decision-making, cognitive control and effortful control could be part of a genetic maturation process. Decision-making [72] and cognitive control [73] are important for developing strategies of action and thus be implied in the persistence or desistance of aggression. Although we did not find studies of its association with PA, effortful control has been related to RA [38, 39] and could partly explain individual differences in the developmental change in RA during childhood.

We found nonshared environmental factors associated with developmental changes of PA and RA, suggesting a common nonshared environmental modulation hypothesis. Nonshared environmental factors have also been found in other studies and could represent effects of affiliation with deviant peers or popularity status [35]. Students who affiliate with deviant peers and students who were more popular tended to engage more in PA. Students who gain social status by using PA could replicate the behavior and thus follow a less steep decline compared to their peers; in turn, conflicts and betrayal among aggressive friends, as well as fluctuations in popularity status, could also foster RA. This explicit proposition of nonshared environmental factors needs to be tested in future studies.

We also found that PA’s baseline level and developmental change are associated with the same shared environmental factors, suggesting a shared environmental set point mechanism specific to PA. This finding suggests that both parameters of the development of PA through childhood could be associated with socialization factors that are not related to the development of RA. The current literature has been inconsistent in regard to shared environmental effect, with some behavioral genetic studies supporting familial effects on PA [52, 56], as others did not [53, 57]. In contrast to past behavioral genetic studies, the present findings support effects of socialization. Shared environmental factors could represent influences such as endorsement of aggressive behavior as an adequate goal-directed behavior [27] or lack of parental discipline and monitoring [29]. As is the case with genetic factors, a genetically informed study with explicit measures of familial process would be needed to precisely assess what exactly is composing these shared environmental factors.

Furthermore, RA’s developmental change is associated with shared environmental factors that are independent of PA’s development, suggesting a specific shared environment modulation hypothesis for RA. Shared environmental factors associated with RA were also found in precedent studies [52, 56] but rarely interpreted. We found two different specific shared environmental factors associated with developmental change in RA. It could indicate that multiple socialization factors act in an additive manner on the development of RA. We suggest that a combination of familial characteristics could explain our finding. For example, lack of parental warmth or neglect [30, 33, 45] could foster the development of RA and have additive effects with social adversity experienced in the family.

Finally, the baseline level of RA is also associated with specific genetic factors, suggesting a genetic differentiation hypothesis specific to RA. This finding suggests that PA and RA could have partly specific genetic underpinnings. RA’s baseline level may be better explained by a combination of characteristics also associated with PA and characteristics specifically associated with RA. Characteristics uniquely associated with RA could be temperamental, like negative emotionality, anxiety or anger, or related to the endocrine system that could be involved in the regulation of RA through its effect on the modulation of impulsivity [44].

### Strengths and limitations

The use of a longitudinal twin design allowed for a description of the development of PA and RA through childhood, extending the few existing studies on this topic. The design was appropriate to detail genetic and environmental factors associated with interindividual differences in baseline levels and developmental change of PA and RA. Our genetic and developmental approach also adopts a novel view on the issue of the correlation between PA and RA by decomposing the association between the two into genetic and environmental factors.

Despite these strengths, our study also has a number of limitations. First, sample size was an issue for identifying significant factors and to consider sex differences. We decided to interpret the results of the bivariate latent growth curve model with Cholesky decomposition even if some loadings on latent factors were not statistically significant. Without a doubt, our findings need to be replicated in larger samples before more definite conclusions can be drawn. Also, the use of teachers as the sole informant imposes a limit on generalizations because, as Baker, Raine (52) showed, estimates of genetic and environmental influences may vary according to the informant (mother or child ratings vs. teachers). Sex differences have also been shown in the Baker et al. study and should be investigated in the study of the overlapping development of Pa and RA.

A limit inherent with twin studies is that it can only estimate the relative magnitude of genetic and environmental factors as well as test for qualitatively different genetic or environmental factors. As such, any measures that are highly influenced by genetics, such as cognitive and temperamental measures, could be interpreted as part of the genetic factors. Shared environmental factors can be comprised of any element that makes the twins more similar than what could be expected by their genetic relatedness. Those are usually interpreted as evidence of familial or neighborhood effects. Lastly, the nonshared environmental factors can be interpreted as influences from experiences unique to each child. Those can be different peer influences, different treatment by teachers or parents or could also be that each child experiences the same environment differently. Another limitation inherent to behavioral genetic studies is that interactions and correlations between environmental factors and genetic factors that are not modeled may increase estimates of the relative contribution of genetic factors or nonshared environmental factors. Yet, both genes by environment correlations (rGE) and genes by environment interactions (GxE) are plausible. An example of rGE would be that genetically influenced temperamental characteristics typical of reactively aggressive children entail specific reactions of the social environment. It could also be that parents and children share genetic factors that are, for example, both associated with PA and a lack of parental discipline. An example of a possible GxE would be that chronically hostile and threatening environments, such as parental punishment or peer victimization, may foster the expression of a genetic disposition toward RA, which may become stronger as children mature. Future longitudinal studies should investigate these probable rGE and GxE processes. There is also a basic assumptions about equal environments which states that MZ twins are not treated more similarly than DZ twins (in which case, the relative importance of genetic factors would be overestimated). Finally, twins are presumed to be comparable to singletons, in which case the results could also be generalized to singletons.

Lastly, by using growth curves to model the development, we make the assumption that a single form of development with variability around the initial level and the slope describes the population.

## Conclusion

Notwithstanding these limitations, our findings support common genetic and nonshared environment etiologies for PA and RA, as well as specific shared environmental etiologies. The social learning theory of PA is partly supported by our findings of genetic factors which could help enrich and nuance the theory of PA’s development. For RA’s development, an enhanced frustration-aggression theory could include genetic determinants as well as shared and nonshared environmental factors. Following evidence of instrumental aggression in infants 6 months of age, future research could aim at adapting scales of defensive aggression in infants. This could help develop novel research on the precise factors common to PA and RA baseline levels or to their development. Interventions aimed at reducing aggression should be adapted to specific subtypes of aggression, by learning to use alternative behaviors rather than PA for goal achievement or to develop anger management strategies for RA. [33, 74]. The specific shared environment etiologies of PA and RA also need more research. Our results indicate few overlapping shared environmental factors making it an important point of research to help distinguish at the beginning of childhood which developmental subtype is more likely to be developed.

## Supporting information

S1 Table Full sample MZ / DZ covariance matrix. (DOCX)

S2 Table Fit statistics of univariate latent growth models and standardized estimates (%) of genetic, shared and nonshared environmental factors in the univariate biometric latent growth curve models. LL: Loglikelihood; AIC: Akaike Criterion Information; BIC: Bayesian Information Criterion; CFI: Comparative Fit Index; RMSEA: Root Mean Square Error of Approximation; A: Genetic factors; C: Shared environment factors; E: Nonshared environment factors.
(DOCX)

S3 Table Unstandardized parameter estimates with 95% confidence interval of the biometric univariate growth curve models. Note: Naming scheme of the parameters: The letter refers to the biometric component, the first number refers to the destination of an arrow, and the last number to the origin of an arrow. For example, a_21_ indicate a link from the 1^st^ genetic component to the 2^nd^ latent variable (here a slope).
(DOCX)

S4 Table Unstandardized parameter estimates with 95% confidence interval of the biometric bivariate growth curve model. Note: Naming scheme of the parameters: The letter refers to the biometric component, the first number refers to the destination of an arrow, and the last number to the origin of an arrow. For example, a_31_ indicate a link from the 1^st^ genetic component to the 3^rd^ latent variable (here, PA’s slope).
(DOCX)

S5 Table Number of complete and incomplete pairs for each time point. (DOCX)

S6 Table Number and proportion of pairs in the same classroom for each year. (DOCX)

S1 Fig Distribution of Proactive Aggression Scales from 6 to 12 years of age. (DOCX)

S2 Fig Distribution of reactive aggression scales from 6 to 12 years of age. (DOCX)

S1 Equation Equations for the multivariate growth curve model. (DOCX)
