# Supplementary material for: Heterogeneity in the development of proactive and reactive aggression in childhood: Common and specific genetic - environmental factors
Source: PLoS One. 2017 Dec 6;12(12):e0188730. doi: 10.1371/journal.pone.0188730 (PMC5718601; doi:10.1371/journal.pone.0188730)
Supplement: S1 Table — (DOCX) [file pone.0188730.s001.docx]

**S1 Table. Full sample MZ / DZ covariance matrix.**

|  | DZ | Twin 1 | | Twin 2 | |
| --- | --- | --- | --- | --- | --- |
| MZ |  | PA | RA | PA | RA |
| Twin 1 | PA | **Part 1. Twin 1 T-matrix** | | **Part 3. DZ Twin B-matrix** | |
|  | RA |  |  |  |  |
| Twin 2 | PA | **Part 2. MZ Twin B-matrix** | | **Part 4. Twin 2 T-matrix** | |
|  | RA |  |  |  |  |

**Part 1. Twin 1 T-matrix**

|  | DZ | Proactive |  |  |  |  | Reactive |  |  |  |  |
| --- | --- | --- | --- | --- | --- | --- | --- | --- | --- | --- | --- |
| MZ |  | 6 y | 7 y | 9 y | 10 y | 12 y | 6 y | 7 y | 9 y | 10 y | 12 y |
| Proactive | 6 y | - | .417 | .159 | .232 | .181 | .627 | .287 | .280 | .207 | .220 |
|  | 7 y | .476 | - | .492 | .332 | .397 | .421 | .539 | .506 | .311 | .392 |
|  | 9 y | .277 | .472 | - | .445 | .283 | .236 | .379 | .626 | .433 | .246 |
|  | 10 y | .125 | .313 | .634 | - | .499 | .226 | .284 | .471 | .639 | .317 |
|  | 12 y | .201 | .384 | .519 | .614 | - | .303 | .333 | .420 | .416 | .526 |
| Reactive | 6 y | .572 | .520 | .397 | .436 | .298 | - | .479 | .389 | .354 | .370 |
|  | 7 y | .318 | .650 | .448 | .310 | .278 | .539 | - | .644 | .473 | .511 |
|  | 9 y | .332 | .468 | .646 | .558 | .436 | .471 | .480 | - | .581 | .425 |
|  | 10 y | .184 | .369 | .634 | .677 | .511 | .403 | .498 | .622 | - | .556 |
|  | 12 y | .065 | .337 | .478 | .410 | .564 | .256 | .386 | .572 | .461 | - |

**Part 2. MZ Twin B-matrix**

|  | Twin 1 | Proactive |  |  |  |  | Reactive |  |  |  |  |
| --- | --- | --- | --- | --- | --- | --- | --- | --- | --- | --- | --- |
| Twin 2 |  | 6 y | 7 y | 9 y | 10 y | 12 y | 6 y | 7 y | 9 y | 10 y | 12 y |
| Proactive | 6 y | .505 | .450 | .283 | .246 | .139 | .358 | .316 | .248 | .265 | .055 |
|  | 7 y | .192 | .364 | .409 | .267 | .358 | .275 | .369 | .277 | .349 | .138 |
|  | 9 y | .083 | .341 | .564 | .331 | .400 | .204 | .365 | .458 | .431 | .288 |
|  | 10 y | .206 | .312 | .490 | .431 | .395 | .236 | .300 | .380 | .421 | .482 |
|  | 12 y | .074 | .195 | .364 | .506 | .433 | .359 | .190 | .227 | .465 | .325 |
| Reactive | 6 y | .282 | .279 | .299 | .324 | .173 | .544 | .306 | .402 | .126 | .265 |
|  | 7 y | .214 | .357 | .388 | .245 | .225 | .414 | .516 | .378 | .361 | .271 |
|  | 9 y | .257 | .401 | .504 | .359 | .300 | .361 | .447 | .651 | .527 | .458 |
|  | 10 y | .217 | .372 | .483 | .486 | .367 | .386 | .447 | .483 | .567 | .469 |
|  | 12 y | -.032 | .181 | .349 | .387 | .353 | .330 | .184 | .327 | .338 | .488 |

**Part 3. DZ Twin B-matrix**

|  | Twin 1 | Proactive |  |  |  |  | Reactive |  |  |  |  |
| --- | --- | --- | --- | --- | --- | --- | --- | --- | --- | --- | --- |
| Twin 2 |  | 6 y | 7 y | 9 y | 10 y | 12 y | 6 y | 7 y | 9 y | 10 y | 12 y |
| Proactive | 6 y | .277 | .174 | .011 | .091 | .189 | .152 | .111 | .037 | .075 | .045 |
|  | 7 y | .244 | .240 | .188 | .160 | .114 | .181 | .144 | .130 | .138 | .170 |
|  | 9 y | .096 | .166 | .187 | .129 | .237 | .094 | .115 | .035 | .087 | .082 |
|  | 10 y | .129 | .174 | .202 | .175 | .188 | .044 | .152 | .184 | .129 | .104 |
|  | 12 y | .056 | -.011 | .127 | .050 | .231 | .081 | .055 | .049 | .056 | .120 |
| Reactive | 6 y | .240 | .187 | .040 | .086 | .112 | .301 | .241 | .169 | .138 | .087 |
|  | 7 y | .188 | .256 | .196 | .251 | .233 | .184 | .264 | .249 | .123 | .187 |
|  | 9 y | .072 | .055 | .206 | .186 | .241 | .111 | .201 | .208 | .178 | .178 |
|  | 10 y | .198 | .171 | .130 | .296 | .258 | .153 | .280 | .249 | .228 | .175 |
|  | 12 y | .069 | .148 | .170 | .264 | .271 | .237 | .193 | .238 | .302 | .267 |

**Part 4. Twin 2 T-matrix**

|  | DZ | Proactive |  |  |  |  | Reactive |  |  |  |  |
| --- | --- | --- | --- | --- | --- | --- | --- | --- | --- | --- | --- |
| MZ |  | 6 y | 7 y | 9 y | 10 y | 12 y | 6 y | 7 y | 9 y | 10 y | 12 y |
| Proactive | 6 y | - | .318 | .286 | .316 | .206 | .631 | .236 | .284 | .165 | .157 |
|  | 7 y | .327 | - | .338 | .281 | .263 | .318 | .653 | .333 | .275 | .388 |
|  | 9 y | .142 | .458 | - | .400 | .292 | .203 | .290 | .568 | .368 | .243 |
|  | 10 y | .159 | .293 | .386 | - | .425 | .237 | .345 | .343 | .587 | .480 |
|  | 12 y | .125 | .425 | .402 | .316 | - | .154 | .261 | .418 | .420 | .548 |
| Reactive | 6 y | .459 | .251 | .165 | .193 | .126 | - | .396 | .311 | .262 | .328 |
|  | 7 y | .255 | .663 | .329 | .314 | .308 | .372 | - | .370 | .366 | .401 |
|  | 9 y | .290 | .313 | .666 | .437 | .257 | .367 | .426 | - | .531 | .488 |
|  | 10 y | .284 | .420 | .399 | .608 | .398 | .233 | .520 | .474 | - | .563 |
|  | 12 y | -.013 | .340 | .526 | .449 | .574 | .235 | .379 | .546 | .446 | - |
