# Supplementary material for: Heterogeneity in the development of proactive and reactive aggression in childhood: Common and specific genetic - environmental factors
Source: PLoS One. 2017 Dec 6;12(12):e0188730. doi: 10.1371/journal.pone.0188730 (PMC5718601; doi:10.1371/journal.pone.0188730)
Supplement: S2 Table — LL: Loglikelihood; AIC: Akaike Criterion Information; BIC: Bayesian Information Criterion; CFI: Comparative Fit Index; RMSEA: Root Mean Square Error of Approximation; A: Genetic factors; C: Shared environment factors; E: Nonshared environment factors. (DOCX) [file pone.0188730.s002.docx]

**S2A Table. Fit statistics for the univariate latent growth models.**

|  |  | LL | AIC | BIC | CFI | RMSEA |
| --- | --- | --- | --- | --- | --- | --- |
| Proactive | Phenotypic growth | -2376.00 | 4824.00 | 4979.48 | .90 | .05 |
|  | Growth w. Cholesky | -2377.29 | 4814.58 | 4944.15 | .91 | .05 |
| Reactive | Phenotypic growth | -2888,24 | 5848.48 | 6003.96 | .93 | .05 |
|  | Growth w. Cholesky | -2889,86 | 5841.72 | 5975.61 | .93 | .05 |

LL: Loglikelihood; AIC: Akaike Criterion Information; BIC: Bayesian Information Criterion; CFI: Comparative Fit Index; RMSEA: Root Mean Square Error of Approximation.

**S2B Table. Standardized estimates of genetic, shared and nonshared environmental factors in the univariate biometric latent growth curve models (%).**

|  | A_1_ | A_2_ | C_1_ | C_2_ | E_1_ | E_2_ |
| --- | --- | --- | --- | --- | --- | --- |
| PA baseline | **68.3** |  | 17.7 |  | *14.0* |  |
| PA change | 6.8 | *57.5* | 21.5 | 0 | 2.4 | 11.7 |
| RA baseline | **78.2** |  | 5.6 |  | **16.2** |  |
| RA change | 1.4 | 2.1 | **38.1** | 9.9 | *9.4* | **39.2** |

We first tested for quadratic trends separatly for each phenotype. In the univariate growth model of PA, the quadratic terms for the MZ (*b*=-.002; *p*=,45) and DZ (*b*=-.004; *p*=,16) groups were not significant and the linear growth model had a slightly better BIC (BIC=5092.49 for the linear model and BIC=5108.55 for the quadratic model). A likelihood ratio test comparing the linear growth model with the more general quadratic growth model also indicated the quadratic growth parameter could be dropped without a significant loss in fit (chisquare=9.2; df=4; *p*=.06). For the univariate growth model of RA, the quadratic terms were significant for both MZ (*b*=-.011; *p*=,001) and DZ (*b*=-.00; *p*=,05) twins. The BIC again indicated a very slight advantage for the linear model (BIC=6160.69) over the quadratic model (BIC=6161.93). The likelihood ratio test suggested that the linear growth model had a significantly different fit compared to the quadratic model (chisquare=24.0; df=4; *p*=.00).

Even if the quadratic trend was significant for RA and close to significant for PA, we chose to use a linear model for few reasons. First, it is worth noting that linear only parameters and linear plus quadratic parameters can sometimes describe very similar trends in data when the curvature of the quadratic function is very long. With a sufficiently big sample, significant quadratic terms may be found even when the trend in the actual observed range is quasi-linear. In the case of RA, although the quadratic terms were significant, the trajectory curve was very shallow, making the trend covering the actual data points quasi-linear. Second, the BIC indicated the model of linear growth had (slightly) better fit compared to the quadratic growth. The BIC is a fit index that penalizes model complexity, it thus indicate that the quadratic term, even if statistically significant, does not produce a significant improvement to the model fit. Third, two of our main objectives needed to be tested with more complex models (multivariate growth and genetic decomposition) which weighted heavily to keep the model parcimonious. Based on the preliminary analyses, and considering our research objectives we selected a linear trend as the best growth model for the two outcome.

Results of the phenotypic latent growth curve model of PA revealed a baseline level significantly different than zero (I_MZ_=.37, CI 95% .32-.43; I_DZ_=.39, CI 95% .34-.44) and a slight decline from 6 to 12 years of age (S_MZ_=-.02, CI 95% -.04--.01; S_DZ_=-.02, CI 95% -.03--.01). A small association between baseline level and systematic change (cov_IS-MZ_=-.01, CI 95% -.02--.001; cov_IS-DZ_=-.01, CI 95% -.02--.002) was also found, but only for MZ pairs. Intraclass correlations of baseline levels of PA were *r* = .85 for MZ and *r*= .52 for DZ, suggesting some shared environment factors associated with the baseline level of PA. For the slope of RA, intraclass correlations were .83 for MZ pairs and .51 for DZ pairs, suggesting genetic and some shared environmental factors. Standardized variance components (i.e. percentages of the overall variance due to each component) from the biometric latent growth curve model of PA are presented in S2B Table (unstandardized parameter estimates can be found in S3 Table of the accompanying supporting information). Genetic factors explain 68.3% of the variance of the baseline level of PA. Shared and nonshared environmental factors were also important, accounting respectively for 17.7% and 14% of the variance in the baseline level of PA. Genetic factors also affected the slope of PA but those were mostly independent from the ones associated with PA’s intercept. As for environmental factors, shared and nonshared environmental factors accounted for 21.5% and 14.1%, respectively, of the slope variance. The shared environmental effects on systematic change were the same factors that also influenced the baseline level of PA. On the other hand, the nonshared environmental factors associated with systematic change in PA were mostly distinct from the ones associated with the baseline level of PA. Overall, these results indicate that the genetic factors associated with the baseline level of PA at age 6 are not associated with systematic change in this behavior from age 6 to age 12. In fact, different genetic factors seem to be involved in systematic change of PA, suggesting two different genetic hypotheses are needed to explain the development of PA in childhood: a genetic continuity hypothesis and a genetic maturation hypothesis. Shared and nonshared environmental factors are also associated with both baseline level and systematic change of PA. The same shared environmental factors seem to be associated with both baseline level and systematic change, supporting a shared environment set point hypothesis. Finally, there are also nonshared environmental factors that are specific to baseline level and to the systematic change of PA, suggesting again two different hypotheses are needed: a first for baseline level and a second for systematic change. It is worth noting that because the residuals are time-specific, the nonshared environmental factors found on the baseline level and systematic change of PA are free of measurement error.

The results from the phenotypic latent growth curve of RA scores showed a baseline level significantly different from zero (I_MZ_=.51, CI 95% .44-.58; I_DZ_=.56, CI 95% .51-.62) and a systematic decline for MZ and DZ pairs from 6 to 12 years of age (S_MZ_=-.02, CI 95% -.04--.004; S_DZ_=-.03, CI 95% -.04--.02) as well as a small nonsignificant covariance between baseline level and slope (cov_IS-MZ_=-.01, CI 95% -.02-.001; cov_IS-DZ_=-.01 CI 95% -.02-0). The intraclass correlations between baseline levels of RA were *r* = .82 for MZ pairs and *r* = .45 for DZ pairs, suggesting strong association with genetic factors and the baseline level of RA at age 6 years. For the systematic change of RA, intraclass correlations were .50 for MZ pairs and .52 for DZ pairs, suggesting strong shared environmental factors. Standardized variance components (i.e. percentages of the overall variance due to each component) from the biometric latent growth curve model of RA are shown in S2B Table and indicate a somewhat simpler story than for PA. The baseline level of RA was largely associated with genetic (78.2%) and nonshared environmental (16.2%) factors. The systematic change of RA was merely associated with environmental factors, both shared (48%) and nonshared (48.6%). These results support a hypothesis of genetic differentiation, as well as hypotheses of shared environment modulation and nonshared environment modulation.
