# Supplementary material for: Heterogeneity in the development of proactive and reactive aggression in childhood: Common and specific genetic - environmental factors
Source: PLoS One. 2017 Dec 6;12(12):e0188730. doi: 10.1371/journal.pone.0188730 (PMC5718601; doi:10.1371/journal.pone.0188730)
Supplement: S3 Table — Note: Naming scheme of the parameters: The letter refers to the biometric component, the first number refers to the destination of an arrow, and the last number to the origin of an arrow. For example, a21 indicate a link from the 1st genetic component to the 2nd latent variable (here a slope). (DOCX) [file pone.0188730.s003.docx]

**S3 Table. Unstandardized parameter estimates with 95% confidence interval for the biometric univariate growth curve models.**

| Phenotype | Biometric component | Parameter | Unstandardized estimate | 95% Confidence interval |
| --- | --- | --- | --- | --- |
| Proactive aggression |  |  |  |  |
|  | Genetic |  |  |  |
|  |  | a_11_ | 0.277 | (0.168 - 0.347) |
|  |  | a_21_ | -0.015 | (-0.042 - 0.023) |
|  |  | a_22_ | 0.045 | (0.000 - 0.059) |
|  | Shared environment |  |  |  |
|  |  | c_11_ | 0.141 | (-0.006 - 0.261) |
|  |  | c_21_ | -0.027 | (-0.051 - 0.026) |
|  |  | c_22_ | 0.000 | (0.000 - 0.000) |
|  | Nonshared environment |  |  |  |
|  |  | e_11_ | 0.125 | (0.022 - 0.186) |
|  |  | e_21_ | -0.009 | (-0.035 - 0.023) |
|  |  | e_22_ | 0.020 | (0.000 - 0.038) |
| Reactive aggression |  |  |  |  |
|  | Genetic |  |  |  |
|  |  | a_11_ | 0.385 | (0.295 - 0.449) |
|  |  | a_21_ | -0.007 | (-0.030 - 0.025) |
|  |  | a_22_ | -0.008 | (-0.056 - 0.000) |
|  | Shared environment |  |  |  |
|  |  | c_11_ | 0.103 | (-0.119 - 0.266) |
|  |  | c_21_ | -0.036 | (-0.062 - 0.002) |
|  |  | c_22_ | 0.018 | (0.000 - 0.056) |
|  | Nonshared environment |  |  |  |
|  |  | e_11_ | 0.175 | (0.076 - 0.256) |
|  |  | e_21_ | -0.018 | (-0.044 - 0.020) |
|  |  | e_22_ | 0.036 | (0.007 - 0.054) |

^1^ Naming scheme of the parameters: The letter refers to the biometric component, the first number refers to the destination of an arrow, and the last number to the origin of an arrow. For example, a_21_ indicate a link from the 1^st^ genetic component to the 2^nd^ latent variable (here a slope).
