# Supplementary material for: Heterogeneity in the development of proactive and reactive aggression in childhood: Common and specific genetic - environmental factors
Source: PLoS One. 2017 Dec 6;12(12):e0188730. doi: 10.1371/journal.pone.0188730 (PMC5718601; doi:10.1371/journal.pone.0188730)
Supplement: S5 Table — (DOCX) [file pone.0188730.s005.docx]

**S5 Table. Number of complete and incomplete pairs for each time point**

Table S5.1. Number of complete pairs with data on 1,2,3,4 or 5 occasions

|  | Proactive | Reactive |
| --- | --- | --- |
| 5 occasions | 120 | 118 |
| 4 | 163 | 164 |
| 3 | 100 | 100 |
| 2 | 96 | 96 |
| 1 | 68 | 69 |
| 0 occasion | 8 | 8 |
| Total | 555 | 555 |

Table S5.2. Number of individual twins and complete pairs for proactive and reactive aggression per year

|  | 6 years | 7 years | 9 years | 10 years | 12 years |
| --- | --- | --- | --- | --- | --- |
| Proactive |  |  |  |  |  |
| Individual twins | 786 | 838 | 754 | 773 | 625 |
| Pairs with data on both twins | 386 | 410 | 361 | 372 | 283 |
| Pairs with data on one twin | 14 | 18 | 32 | 29 | 59 |
| Pairs with no data | 155 | 127 | 162 | 154 | 213 |
| Reactive |  |  |  |  |  |
| Individual twins | 783 | 838 | 753 | 772 | 625 |
| Pairs with data on both twins | 383 | 410 | 360 | 371 | 283 |
| Pairs with data on one twin | 17 | 18 | 33 | 30 | 59 |
| Pairs with no data | 155 | 127 | 162 | 154 | 213 |
