# Supplementary material for: Heterogeneity in the development of proactive and reactive aggression in childhood: Common and specific genetic - environmental factors
Source: PLoS One. 2017 Dec 6;12(12):e0188730. doi: 10.1371/journal.pone.0188730 (PMC5718601; doi:10.1371/journal.pone.0188730)
Supplement: S6 Table — (DOCX) [file pone.0188730.s006.docx]

**S6 Table. Number and proportion of pairs in the same classroom for each year**

|  | 6 years | | 7 years | | 9 years | | 10 years | | 12 years | |
| --- | --- | --- | --- | --- | --- | --- | --- | --- | --- | --- |
|  | N | % | N | % | N | % | N | % | N | % |
| Same class | 119 | 30.3 | 95 | 22.8 | 89 | 22.6 | 109 | 28.7 | 151 | 39.5 |
| Different classes | 274 | 69.7 | 321 | 77.2 | 304 | 77.4 | 271 | 71.3 | 231 | 60.5 |
