# Supplementary figures and images for: Heterogeneity in the development of proactive and reactive aggression in childhood: Common and specific genetic - environmental factors
Source: PLoS One. 2017 Dec 6;12(12):e0188730. doi: 10.1371/journal.pone.0188730 (PMC5718601; doi:10.1371/journal.pone.0188730)

**S1. Distribution of Proactive Aggression Scales from 6 to 12 years of age.**


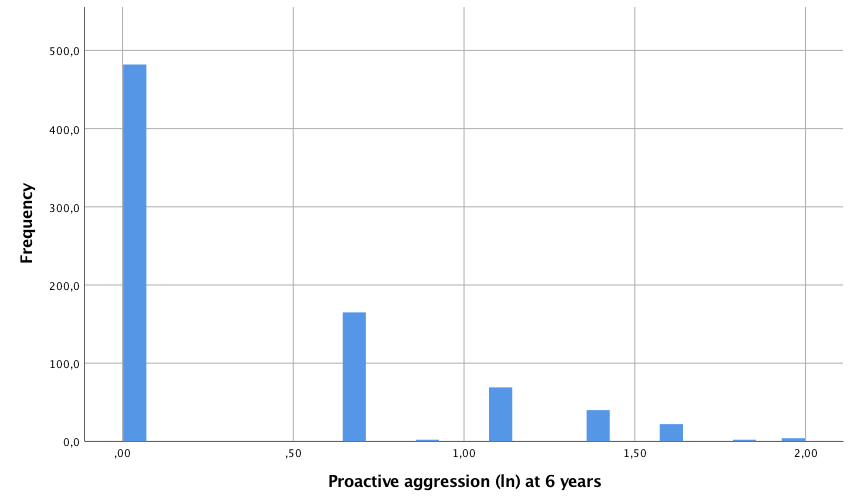


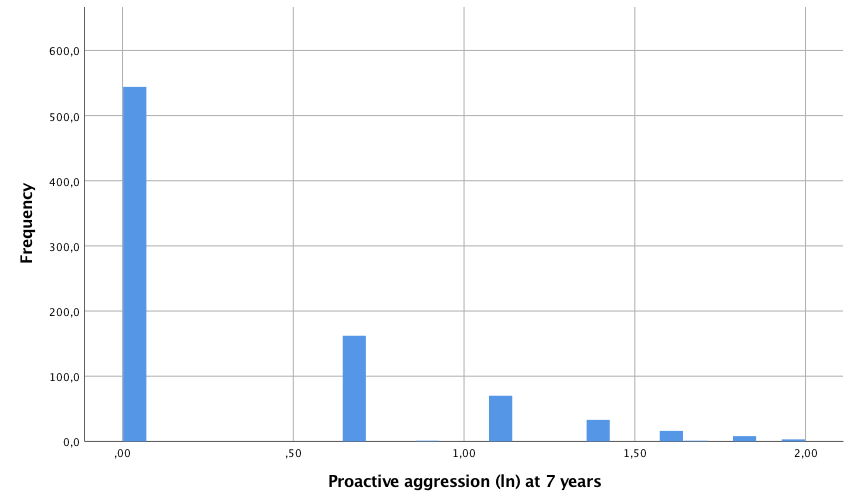


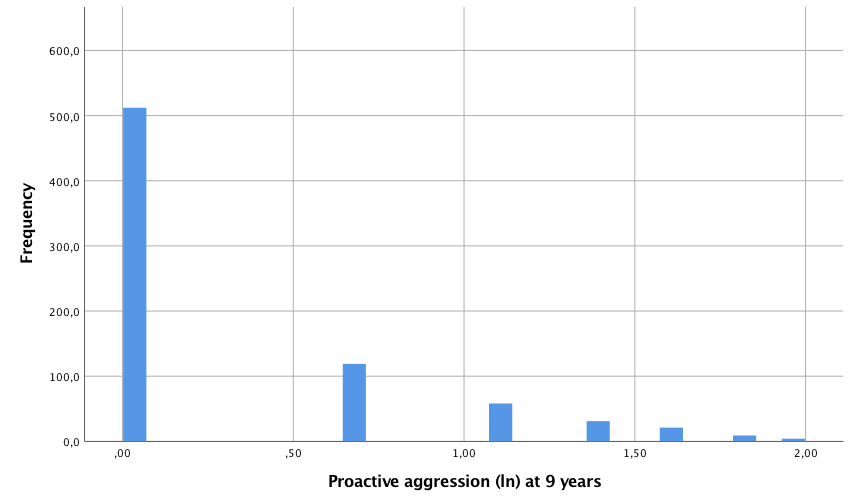


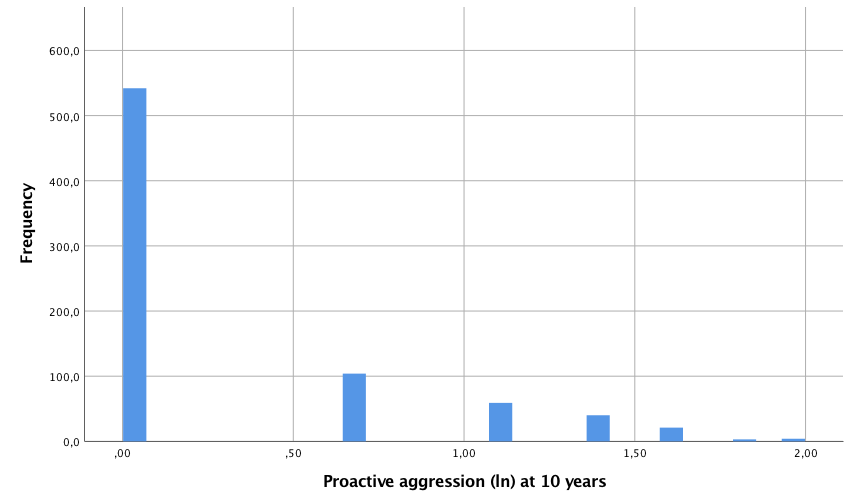


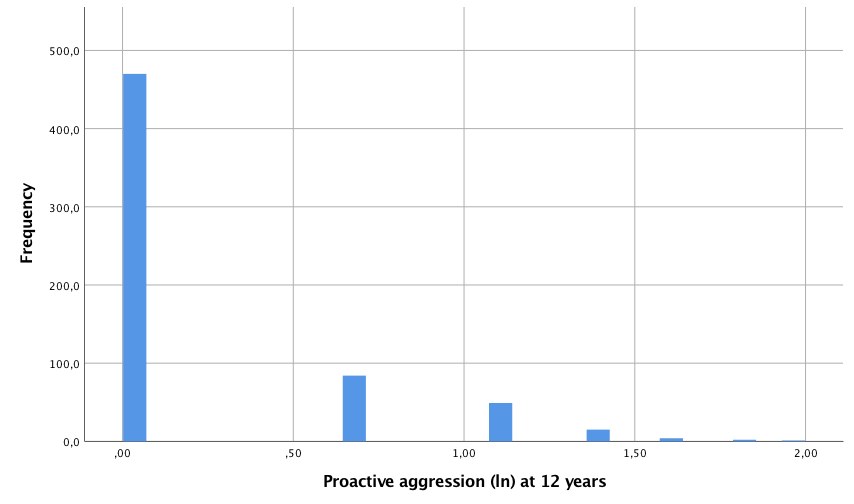

Supplement: S1 Fig — (DOCX) [file pone.0188730.s007.docx]

**S2 Figures. Distribution of Reactive Aggression Scales from 6 to 12 years of age.**


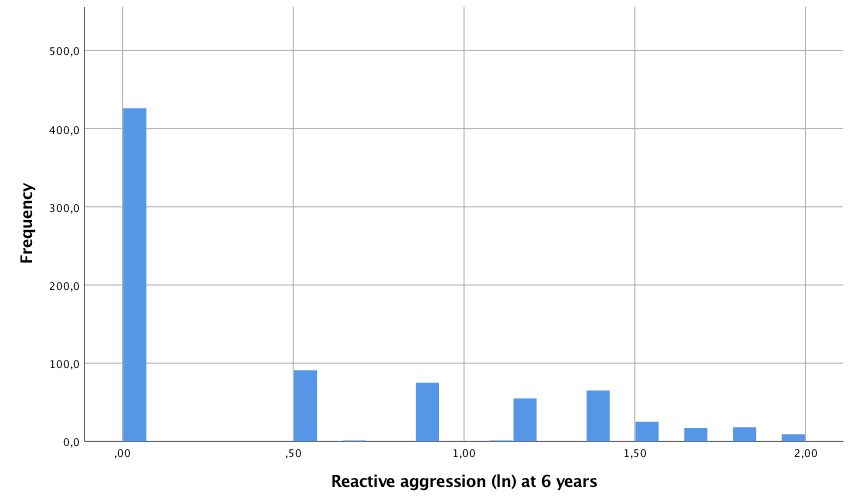


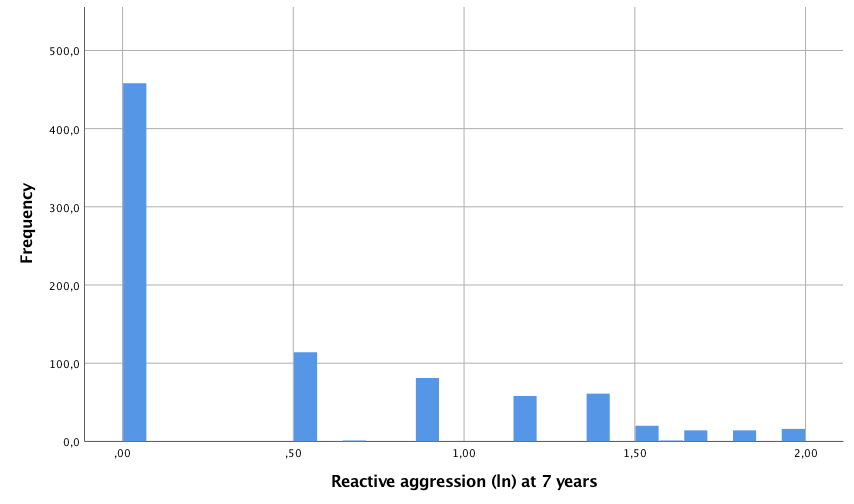


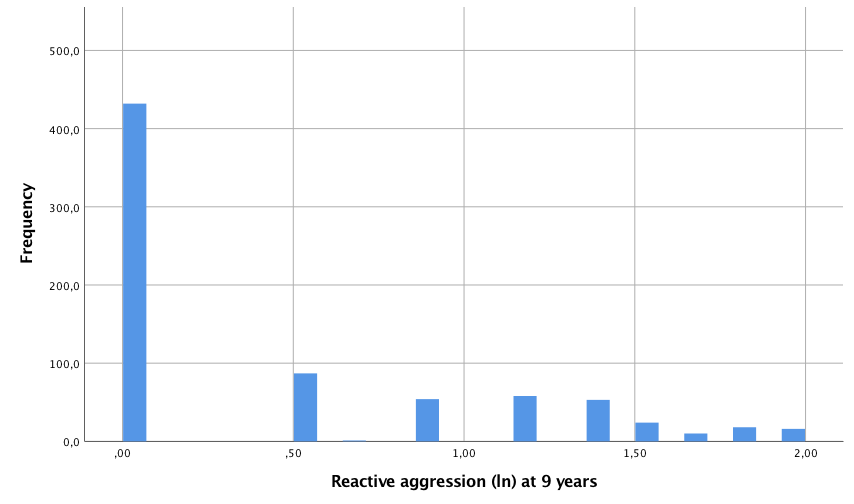


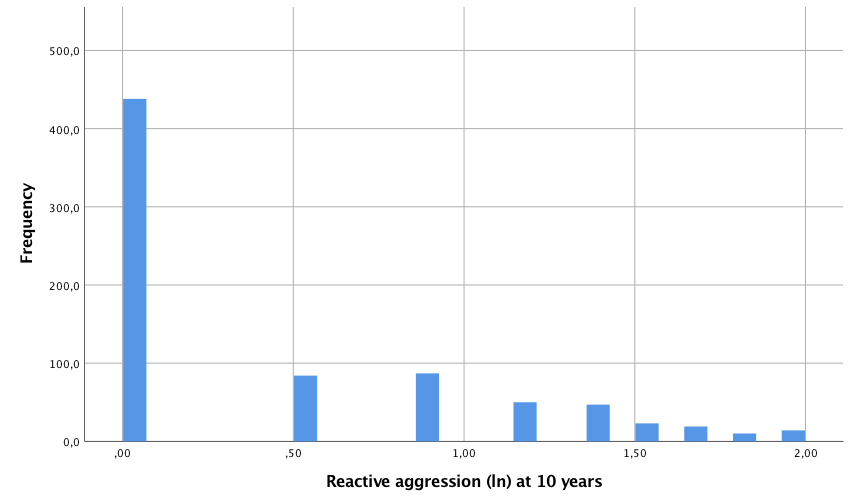


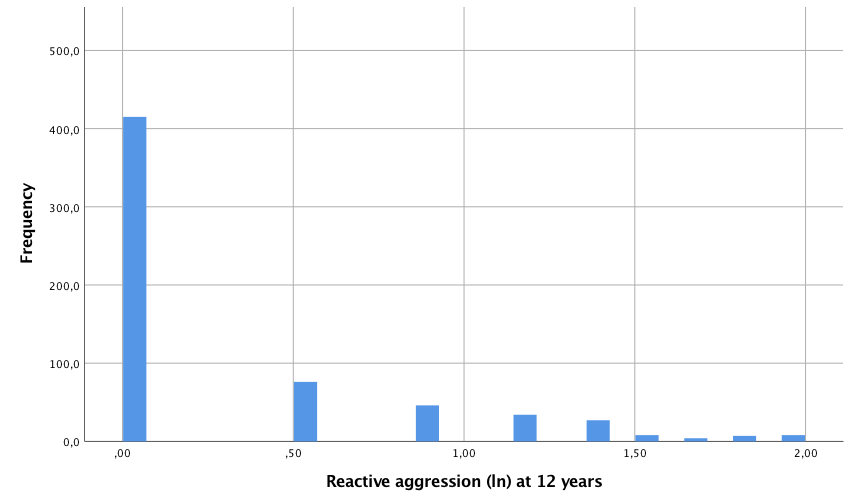

Supplement: S2 Fig — (DOCX) [file pone.0188730.s008.docx]
