# Supplementary material for: Heterogeneity in the development of proactive and reactive aggression in childhood: Common and specific genetic - environmental factors
Source: PLoS One. 2017 Dec 6;12(12):e0188730. doi: 10.1371/journal.pone.0188730 (PMC5718601; doi:10.1371/journal.pone.0188730)
Supplement: S1 Equation — (DOCX) [file pone.0188730.s009.docx]

S9 Equations. Equations for the multivariate growth curve model
